# Current situation of shared decision making in osteoporosis: A comprehensive literature review of patient decision aids and decision drivers

**DOI:** 10.1002/hsr2.849

**Published:** 2022-11-21

**Authors:** Xavier Nogués, María Cristina Carbonell, Laura Canals, Luis Lizán, Santiago Palacios

**Affiliations:** ^1^ Internal Medicine Department, Instituto de investigación hospital del Mar (IMIM)—Centro de Investigación Biomédica en Red de Fragilidad y Envejecimiento Saludable (CIBERFES) Universitat Autonòma de Barcelona Barcelona Spain; ^2^ Department of Medicine, Atenció Primària Barcelona—Institut Català de la Salut (ICS), Grupo GREMPAL Universidad de Barcelona Barcelona Spain; ^3^ Department of Medicine Amgen Europe Risch‐Rotkreuz Switzerland; ^4^ Department of Outcomes Research Outcomes'10 Castellón de la Plana Spain; ^5^ Department of Medicine Universitat Jaume I Castellón de la Plana Spain; ^6^ Instituto Palacios Salud de la Mujer Madrid Spain

**Keywords:** osteoporosis, patient decision aids, review, share decision‐making

## Abstract

**Background and Aims:**

Osteoporosis is a systemic skeletal disease characterized by low bone mass and microstructural deterioration of bone tissues, resulting in bone fragility and increased fracture risk. It is the most common bone‐related disease in the population. However, the proportion of patients who start treatment but discontinue it during the first year is very high (around 50%). Endeavors are made to promote patient participation in treatment by implementing patient decision aids (PDA), whose function is to help the patient make disease‐related decisions. We aim to summarize the characteristics of the currently available PDA for osteoporosis, as well as deciding factors.

**Methods:**

Comprehensive review of the literature.

**Results:**

Currently, eleven PDAs can be found for osteoporosis. These PDA have different characteristics or options such as information about treatments tailored to patient needs, graphic information of the results (to facilitate understanding), personal histories (learning), tests to check the knowledge acquired, provision of evidence, clinical practice guidelines or a final summary to share with their doctor. Only five of these PDAs can be considered complete since they provide relevant disease information and therapeutic options to the patient, promote patient's reflection and foment patient‐physician discussion.

**Conclusions:**

This study provides an update on the current state of decision making on osteoporosis and available PDA, which can help engage the patient through shared decision‐making by considering, among other things, patient preferences. Physicians should consider PDA, as it may promote adherence and effectiveness of treatment.

## 1 BACKGROUND AND AIMS

Osteoporosis is a systemic skeletal disorder characterized by low bone mass and microarchitectural deterioration of bone tissue that leads to bone fragility and increased fracture risk.^1^, ^2^ It is the most common bone disease affecting predominantly women.^1^, ^3^ About 27 million people are affected by the disease in the European Union (EU)^4^ and, moreover, its prevalence is expected to rise in the coming years.^5^

Since osteoporosis is a silent disease without any pathognomonic clinical signs,^6^ it usually remains undiagnosed until a low‐trauma fracture occurs.^1^ Osteoporotic fracture, commonly involving the hip and spine, may affect an individual's ability to function independently, leading to chronic pain and prolonged rehabilitation.^7^ It is widely recognized that osteoporosis‐related fractures are associated with increased mortality, with the exception of forearm fractures.^8^ Thus, between 20% and 40% of individuals suffering hip fractures die within a year.^9^

The economic burden of osteoporosis is also high. In the EU it is estimated that the cost of osteoporotic fractures in 2010, including pharmacological and long‐term disability, reached 37,000 million euros.^10^, ^11^

### 1.1 Strategies to prevent osteoporosis fractures

To prevent fractures in osteoporotic patients, both nonpharmacological and pharmacological interventions are recommended. Nonpharmacological strategies include a healthy lifestyle, such as a balanced diet, regular physical exercise, no smoking, limited alcohol consumption, and implementation of fall prevention measures.^1^, ^12^

Pharmacological treatments, such as antiresorptive and anabolic drugs, target patients with high or very high fracture risk. Both therapies have been shown to increase bone strength.^2^, ^13^ However, their mechanisms of action differ, as antiresorptive therapies inhibit bone resorption by suppressing osteoclastic‐mediated bone breakdown and bone turnover, whereas anabolic agents restore bone mineral content.^2^, ^14^, ^15^ Main pharmacological agents currently available for osteoporosis are: bisphosphonates, anti‐RANKL antibodies (denosumab), selective estrogen receptor modulators, estrogen replacement, monoclonal antisclerostin antibodies (romosozumab), strontium ranelate and parathyroid hormone analogs.^16^, ^17^ The latest updates in clinical practice guidelines recommend teriparatide, abaloparatide, or romosozumab for patients with a very high risk of osteoporosis fracture.^17^, ^18^

Despite the wide range of pharmacological options available, there is a large gap between the number of women receiving treatment and those that could be considered eligible for treatment based on their fracture risk.^19^ Several studies reveal that less than 20% of patients suffering from a fragility fracture receive therapy to reduce future fractures within the following year.^19^ Moreover, many women at high risk of fractures choose not to initiate therapy, and, of those who do, up to 50% discontinue treatment in less than 1 year.^20^ These low rates of treatment compliance and persistence fail to reduce the risk of osteoporotic fractures, which in turn can increase healthcare costs and greatly decrease patients' quality of life^21^ and life expectancy.^8^, ^22^

Previous studies have shown that patient preferences play a key role in accepting or rejecting osteoporosis treatment.^23^ Accordingly, in addition to choosing the treatment based on the patient's characteristics and their risk of fractures,^7^ it is fundamental to align treatment choice with patients' preferences and engage them in treatment decisions.

### 1.2 Patient‐centered care and patient decision aids

In broad terms, there are three models of doctor‐patient interaction regarding clinical decisions: (1) *paternalism*, where the physician has all the relevant information and is the sole decision maker; (2) *informed model*, where the physician presents “the facts” and the patient makes all decisions; and (3) *shared decision‐making* (SDM), where the physician and patient share information, discuss options using the best evidence and reach a collaborative decision that takes into account the patient's context, values, and preferences.^24^, ^25^, ^26^, ^27^

The SDM is a key component of patient‐centered care. In this model, the physician offers a recommendation and shares information about the benefits, drawbacks, and burdens of the therapeutic options available. At the same time, patients are encouraged to become involved in the decision and to express their feelings and treatment expectations.^25^, ^26^, ^28^ This process encompasses five steps: (1) understanding the patient's experience, preferences, and expectations; (2) building partnerships; (3) providing treatment evidence, including uncertainties; (4) giving recommendations, and (5) checking for understanding and agreement.^25^ SDM does not advise the patient to choose one option over another, it provides structured guidance in the decision‐making steps and helps patients to make informed value‐based decisions together with their physician.^29^, ^30^ The final choice depends on how a patient evaluates the drawbacks and benefits of the different treatment options.^31^ In 1989 a paper was published describing clinical strategies when a patient has decisional needs.^32^ Subsequently, in 1995 the Patient Decision Aids Research Group developed the Decisional Conflict Scale, the first scale to measure changes in decisional requirements following counseling.^33^ In the following years, different guidelines for developing PDAs were published. Also, in 2003 the International Patient Decision Aid Standards (IPDAS) was founded. It aimed to improve the quality and effectiveness of patient decision aids by establishing a shared evidence‐based framework with a set of criteria to improve their content, development, implementation, and evaluation. In this sense, IPDAS established a set of quality criteria for PDA that works as a checklist for developers and users^29^, ^30^

On the other hand, the European League Against Rheumatism and the European Federation of National Associations of Orthopaedics and Traumatology  recommend the inclusion of patient education programs to prevent fractures.^20^

In this respect, PDAs play a key role in providing evidence‐based information about conditions, treatment options, outcomes, probabilities, and an opportunity for patients to ponder their preferences.^20^ PDA facilitate SDM by improving the patient's knowledge of the disease and encouraging reflection, generating more realistic expectations about options, reducing decisional conflict, and helping patients to clarify their preferences.^26^, ^27^, ^34^ PDA also help patient to share information with the physician, allowing a consensus to be reached on the best treatment option.^24^

Although further research is required to determine the effect of adherence, involving patients in decision‐making could lead to improved disease management.^35^, ^36^ This becomes particularly relevant when there is more than one therapeutic option, each with its benefits and drawbacks, as is the case for osteoporosis. Previous studies have shown that the use of PDA contributes to reducing decisional conflict (i.e., uncertainty about the choice, ignorance about the pros and cons of each option, pressure to make a particular choice, and effectiveness of the decision).^37^, ^38^, ^39^, ^40^, ^41^ In addition, the use of a PDA can also contribute to reducing the variation of the clinical practice in preference‐sensitive options, and improve care more broadly.^27^, ^28^, ^30^, ^34^

This study aims to summarize the characteristics of the currently available PDA for osteoporosis and decision factors.

## 2 METHODS

Two literature reviews were conducted: (1) a review of studies on physician and patient preferences for osteoporosis treatments; and (2) an ordered review of the literature on PDA for patients with Osteoporosis.

### 2.1 Data sources and search strategies

The international databases PubMed/Medline and Cochrane library, and the national Medicina en Español (MEDES) and the Índice Bibliográfico Español en Ciencias de la Salud (IBECS) databases, were searched to identify relevant publications on treatment preferences. PubMed/Medline and manual sources (e.g., google, google scholar, Ottawa Hospital) were searched to identify available PDAs for Osteoporosis.

Databases were searched using both MeSH (Medical Subject Headings) and free‐text terms, combined with the Boolean connectors “OR” and “AND.” The treatment preferences search was conducted in English (international database) and Spanish (national database). International database search strategies: (“osteoporosis”) AND (“treatment” OR “management” OR “drug therapy” OR “medication” OR “patient‐centered” OR “shared decision making”) AND (“conjoint analysis” OR “DCE” OR “discrete choice” OR “decision aid” OR “decisional conflict” OR “preference” OR “trade‐off” OR “risk‐benefit” OR “willingness to pay” OR “WTP” OR “willingness to accept”). National database search strategies: “Osteoporosis” AND “preferencias” AND (“tratamiento” OR “manejo” OR “farmacoterapia” OR “medicación”). The search to identify available PDA was conducted as follows: (“Osteoporosis”[Mesh] OR “Osteoporosis” [All Fields]) AND (“Decision Support Techniques” [Mesh] OR “Decision Support Techniques” [All Fields] OR “Decision Aids” [All Fields] OR (“Decision” [All Fields] AND “support” [All Fields] AND “Techniques” [All Fields]) OR (“Decision” [All Fields] AND “Aids” [All Fields])).

### 2.2 Eligibility criteria

Clinical trials, observational studies, and narrative or systematic reviews assessing the preferences for osteoporosis treatment attributes of patients with Osteoporosis and/or the healthcare professionals responsible for their management were included in the systematic search. They needed to be conducted in Europe or North America and published in English or Spanish between 1 January 2008 and 18 December 2018. The second search included original articles and reviews providing data on AID for osteoporosis published in English or Spanish before 20 December 2018.

## 3 RESULTS AND DISCUSSION

We identified 619 records which resulted in the inclusion of 32 studies. A total of 26 of them were related to physician and patient preferences on osteoporosis treatments and 12 PDA for patients with osteoporosis.

### 3.1 PDA

Eleven PDAs are currently available worldwide for osteoporosis. IPDAS establish a set of quality criteria for PDA that works as a checklist for developers and users The IPDAS criteria provided by each PDA are detailed in Table 1. On the other hand, considering that a complete PDA explains the decision; provides information on options, benefits, and harms; and helps patients clarify which benefits and harms matter most, only five of the identified tools can be considered as complete PDA, since they are the only ones that provide information, explore patients' preferences and facilitate physician‐patient discussion (Table 1).^41^, ^42^, ^43^, ^44^, ^45^

**Table 1:** IPDAS criteria

			Patient decision aids						
		Indicadores IPDAS	No. 3	No. 6	No. 7	No. 8	No. 9	No. 10	No. 11
Content	1	Describe the health condition		X	X	X	X	X	X
	2	List the options	X	X	X	X	X	X	X
	3	List the options of doing nothing	X	X		X	X	X	X
	4	Describe the natural course without options	X	X	X	X	X	X	X
	5	Describe procedures		X		X	X	X	X
	6	Describe positive features [benefits]	X	X	X	X	X	X	X
	7	Describe negative features of options [harms/side effects/disadvantages]	X	X	X	X	X	X	X
	8	Include chances of positive/negative outcomes	X				X	X	X
	9	Use event rates specifying the population and time period	X	X	X		X	X	
	10	Compare outcome proabilities using the same denominator, time period, scale	X	X	X		X	X	X
	11	Describe uncertainty around probabilities			X		X	X	X
	12	Use visual diagrams	X	X	X		X	X	X
	13	Use multiple methods to view probabilities		X					
	14	Allows the patient to select a way of viewing probabilities [words, numbers, diagrams]							
	15	Allow patient to view probabilities based on their own situation	X						
	16	Place probabilities in context of other events							
	17	Use both positive and negative frames	X	X	X	X	x	x	x
	18	Describe the procedures and outcomes to help patients imagine what it is like to experience their physical, emotional, social effects	X	X	X	X	X	X	X
	19	Ask patients to consider which positive and negative features matter most				X	X	X	X
	20	Suggest ways for patients to share what matters most with others							
	21	Provide steps to make a decision		X	X				
	22	Suggest ways to talk about the decision with a health professional	X	X		X	X	X	X
	23	Include tools to discuss options with others			X	X	X	X	X
Development	1	Able to compare positive/negative features of options	X	X	X	X	X	X	X
	2	Shows negative/positive features with equal detail		X	X	X	X	X	X
	3	Includes developers' credentials/qualifications		X	X	X			
	4	Finds out what users [patients/practitioners] need to discuss options.		X	X	X			
	5	Has peer review by patient/professional experts not involved in development and field testing		X	X	X			
	6	Is field tested with users			X	X	X	X	X
	7	The field tested with users [patients, practitioners] show the patient decision aid is: acceptable			X	X			
	8	The field tested with users [patients, practitioners] show the patient decision aid is: balanced for undecide patients			X	X			
	9	The field tested with users [patients, practitioners] show the patient decision aid is: understood by those with limited reading skills							
	10	Provides references to evidence used	X	X	X	X	X	X	X
	11	Report steps to find, appraise, summarize evidence							
	12	Report date of last update	X	X	X	X	X	X	X
	13	Report how often patient decision aid is updated							
	14	Describe quality of Scientific evidence [including lack of evidence]							
	15	Use evidence from studies of patients similar to those of target audience							
	16	Report source of funding to develop and distribute the patient decision aid	X	X	X	X			
	17	Report weather authors or their affiliations stand to gain or lose by choices patient make after using the PDA		X	X	X	X	X	X
	18	Is written at a level that can be understood by the majority of patients in the target group	X	X	X	X			
	19	Is written at a grade 8 equivalent level or les according to readability score [SMOG o FRY]							
	20	Provides ways to help patients understand information other tan reading [audio, video, in‐person discussion]	X						
	21	Provide a step‐by‐setp way to move through the web pages)	X						
	22	Allow patients to search for key words							
	23	Provide feedback on personal health information that is entered into the patient decision aid							
	24	Provides security for personal health information entered into the decision aid							
	25	Make it easy for patients to return to the decision aid after lining to other web pages							
	26	Permit printing as a single document				X			
	27	Use stories that represent a range of positive and negative experiences							
	28	Reports if there was a financial or other reason why patients decided to share their story							
	29	State in an accessible document that the patient gave informed consent to use their stories							
Efectividad	1	Recognize a decision needs to be made	X						
	2	Know options and their features	X		X	X			
	3	Understand that values affect decision							
	4	Be clear about option features that matter most							
	5	Discuss values with their practitioner							
	6	Become involved in preferred ways	X						
	7	Improves the match between the chosen option and the features that matter most to the informed patient			X				

*Note*: 3. Osteoporosis Choice; 6. Bisphosphonates for treating osteoporosis; 7. Osteoporosis Decision Support Tool; 8. Osteoporosis: Should I Take Bisphosphonate Medicines?; 9. Should I take alendronate (Fosamax®) for osteoporosis?; 10. Should I take Etidronate (Didronel®) for osteoporosis?; 11. Should I take risedronate (Actonel®) for osteoporosis?

Abbreviation: IPDAS, International Patient Decision Aid Standards.

Concerning the treatment options, some of the PDA support patients in the decision to choose a specific drug.^36^, ^37^, ^38^, ^39^, ^41^, ^42^, ^43^, ^44^, ^45^, ^46^, ^47^ Most tools are focused on bisphosphonate treatment while only three display most of the available therapies. In relation to the format, four of the PDA are on paper,^37^, ^43^, ^44^, ^45^ four on online platforms^36^, ^39^, ^41^, ^47^, ^48^ and three are in both formats.^38^, ^42^, ^46^ These PDAs have different target users according to gender, age, osteoporosis or osteopenia diagnosis, menopausal status, and/or history of fractures. All of them target the English‐speaking population (Table 2).

**Table 2:** Available PDA for osteoporosis

Decision‐making support tool	Country	Target population	Treatment options	α‐Test	β‐Test	Format	Complete tool^a^
Smallwood et al. (2017)^39^	USA	Postmenopausal women with osteoporosis	Different options (Alendronate, Risedronate, Ibandronate, Zoledronic acid, Denosumab, Raloxifene, Teriparatide)	Yes	Yes	Web	Yes
Hiligsmann et al. (2016)^36^	Netherlands	Postmenopausal women with osteoporosis	Different options (Alendronate, Risedronate, Ibandronate, Zoledronic acid, Denosumab, Raloxifene, Ranelate)	Yes	No	Web and paper	No
Osteoporosis Choice^34^, ^37^	USA	Patients >45 years old with osteopenia or osteoporosis	One option (Bisphosphonates vs none)	Yes	Yes	Web	No
Chess‐Mab^46^	USA	Peri‐ and postmenopausal women	No treatment options	Yes	Yes	Web	No
Cranney et al. (2002)^35^	Canada	Postmenopausal women with osteoporosis	Different options (Alendronate, Etidronate, Hormonal, Raloxifene, Vitamin D/Calcium, Lifestyle)	Yes	Yes	Paper	No
Bisphosphonates for treating osteoporosis^45^ (https://www.nice.org.uk/guidance/ta464/resources)	UK	Osteoporosis patients	Bisphosphonates vs. none	NA	NA	Web and paper	No
Osteoporosis Decision Support Tool^44^ (https://www.healthdecision.org/tool.html#/tool/osteoporosis)	USA	Osteoporosis patients	Bisphosphonates vs. none	NA	NA	Web	No
Osteoporosis: Should I take Bisphosphonate medicines?^40^ (https://healthlinkbc.ca/health-topics/te7592)	USA	Postmenopausal women with osteoporosis	Bisphosphonates vs. healthy lifestyle	NA	NA	Web and paper	Yes
Should I take alendronate (Fosamax®) for osteoporosis?^41^(https://musculoskeletal.cochrane.org/decision-aids)	Canada	Postmenopausal women with osteoporosis and recent previous fracture	Alendronate vs. none	NA	NA	Paper	Yes
Should I take etidronate (Didronel®) for osteoporosis^42^(https://musculoskeletal.cochrane.org/decision-aids)	Canada	Postmenopausal women with osteoporosis and recent previous fracture	Etidronate vs. none	NA	NA	Paper	Yes
Should I take risedronate (Actonel®) for osteoporosis^43^(https://musculoskeletal.cochrane.org/decision-aids)	Canada	Postmenopausal women with osteoporosis and recent previous fracture	Risedronate vs. none	NA	NA	Paper	Yes

Abbreviations: NA, not available; PDA, patient decision aids; UK, United Kingdom; USA, United Sates of America.

^a^ Complete Decision‐making support tool, which must provide relevant disease information and therapeutic options to the patient, promote patient's reflection and foment patient‐physician discussion.

During its development, a PDA should be validated by conducting an alpha and/or beta test. An alpha test is an acceptability test that aims to identify all possible issues before launching a product to users, while a beta test is a user utility test in which a sample of the intended audience tries the product out.^49^, ^50^ Only some of the identified PDA have undergone alpha^37^, ^38^, ^39^, ^41^, ^48^ and/or beta tests^37^, ^39^, ^41^, ^48^ (Table 2).

Identified PDA have different characteristics regarding their adaptability to patient profiles, graphic design, inclusion of personal stories, availability of patient knowledge tests, level of evidence of the available information in the PDA, explicit information about recommendations of clinical practice guidelines, and a final summary to share with the physician (Table 3).

**Table 3:** PDA characteristics

	Tools										
Tools strengths	No. 1	No. 2	No. 3	No. 4	No. 5	No. 6	No. 7	No. 8	No. 9	No. 10	No. 11
Adaptable	×	×	×			×	×				
Graphic design		×	×			×	×		×	×	×
Level evidence information							×		×	×	×
Personal stories								×			
Knowledge test				×				×	×	×	×
Patient preferences	×						×	×	×	×	×
Guides recommendation						×	×				
Final summary							×	×			

*Note*: 1. Smallwood et al. (2016)^39^; 2. Hiligsmann et al. (2016)^36^; 3. Osteoporosis Choice; 4. Chess‐Mab; 5. Cranney et al. (2002)^35^; 6. Bisphosphonates for treating osteoporosis; 7. Osteoporosis Decision Support Tool; 8. Osteoporosis: Should I Take Bisphosphonate Medicines?; 9. Should I take alendronate (Fosamax®) for osteoporosis?; 10. Should I take Etidronate (Didronel®) for osteoporosis?; 11. Should I take risedronate (Actonel®) for osteoporosis?

Abbreviation: PDA, patient decision aids.

Since patient profile in osteoporosis is variable, adaptability is a valued characteristic in PDA. Five^36^, ^38^, ^39^, ^41^, ^46^, ^47^ of the eleven PDA identified are adaptable to patient profile by, i.e., calculating fracture risk within 10 years using FRAX®.^51^

Patients appreciate graphic information about their risk of developing a certain disease or the risk/benefits of a therapeutic option.^52^ Moreover, the use of pictographs may improve the comprehensibility and desirability of the information presented. Seven of the PDAs identified include graphic information.^36^, ^38^, ^39^, ^43^, ^44^, ^45^, ^46^, ^47^

Personal stories about health experiences are used as an established resource in education as a way for patients to learn about diseases and treatments and, are included in some PDA. Although there is insufficient evidence examining whether or not personal stories contribute to the effectiveness of PDA, they were identified as having four functions: (1) to provide factual information to help patients understand the option and their outcomes; (2) to demonstrate how patients value decisions differently; (3) to share a range of options and (4) to exemplify the steps others have taken to reach a decision.^53^ Only one of the PDAs includes patient stories.^42^

The option of performing a patient knowledge test helps evaluate whether the patient has understood the information provided in the PDA related to the disease and its treatment. Five PDA includes a test that evaluates the knowledge acquired by patients.^42^, ^43^, ^44^, ^45^, ^48^

The level of evidence provided for the information presented in the PDA demonstrates the validity and integrity of that information. Four PDAs include an acceptable level of evidence for the information provided.^43^, ^44^, ^45^, ^47^

Clinical practice guidelines provide evidence‐based recommendations founded on rigorous systematic reviews and synthesis of published research in academic, governmental, and private sectors.^27^ Knowing the recommendations of the clinical practice guidelines can help patients to increase their knowledge about the treatment of the disease and, through SDM, individualize these recommendations to the preferences and characteristics of each patient. Two PDA include clinical practice guidelines.^46^, ^47^

Last, the final summary, a synopsis to share preference information with the physician, is an essential part of the PDA since it may facilitate communication with the physician and improve the SDM process. Two PDAs include a brief final summary to share with the physician.^42^, ^47^

It is very important for osteoporosis patients to adopt healthy lifestyles, including a varied and balanced diet, which guarantees the supply of essential nutrients for bone health and the amelioration of osteoporosis.^54^ One of the best ways to build and maintain healthy bones is through exercise. Exercise improves disequilibrium and reduces the risk of falls.^55^ None of the PDA available includes adaptations to the patient's exercise program, which should address flexibility, strength, core stability, cardiovascular fitness, and equilibrium.

Patients expressed a positive attitude towards the use of these PDAs as they improved their preparation for decision‐making and decreased decisional conflict. These PDAs improved knowledge transfer and patient involvement in decision‐making with adequate patient and physician satisfaction, but with a weak or null effect on medication adherence.^36^, ^39^ Interestingly the effectiveness of four PDAs has been evaluated. In three studies,^36^, ^39^, ^41^ decisional conflict scores were found in the PDA arms compared to the conventional decision, and one reported a statistically significant difference.^41^ Moreover, two studies exploring PDAs found a significant difference in the percentage of patients correctly identifying their risk category postintervention^36^, ^39^ and a significant difference when measuring the specific knowledge. Finally, the impact of PDA on adherence was also observed in one study, where more patients‐initiated treatment in the intervention arm compared with controls.

## 4 DECISION DRIVERS

PDA brings various important patient‐centered issues (i.e., mode of administration, side effects, cost) to the foreground, serving as an invitation for the patient and physician to address these together. This means the main decision drivers in PDA are mandatory to ensure efficacy.

Several treatment characteristics that could influence the decision‐making process have been described in the literature: (1) route and frequency of administration^52^, ^56^, ^57^, ^58^, ^59^, ^60^, ^61^, ^62^, ^63^, ^64^, ^65^, ^66^, ^67^, ^68^, ^69^, ^70^, ^71^, ^72^; (2) efficacy^52^, ^57^, ^58^, ^60^, ^61^, ^65^, ^67^, ^69^, ^71^, ^73^; (3) cost of treatment (for the patient and for society)^52^, ^58^, ^62^, ^65^, ^67^, ^71^; (4) adverse events or side effects^52^, ^57^, ^58^, ^60^, ^61^, ^63^, ^64^, ^65^, ^67^, ^69^, ^71^, ^72^, ^73^, ^74^, ^75^; (5) total duration of the treatment^60^; (6) convenience (i.e., number of pills, independency, dosing facility, body position)^57^, ^63^, ^64^, ^74^, ^75^, ^76^, ^77^; (7) food interactions^71^, ^72^; (8) period of treatment availability in the market^58^, ^71^; (9) sequential therapy^58^; (10) drug‐site administration^58^, ^62^; (11) generic or trademark treatment^58^; (12) monotherapy or combination therapy^58^; (13) action mode of the treatment^58^; and (14) general satisfaction.^63^

From the patients' point of view, route and frequency of treatment administration are one of the most important treatment characteristics. In general, patients prefer less frequent administration, even if subcutaneous administration is required since simpler dosage regimens improve lifestyle.^57^, ^67^, ^68^, ^69^, ^73^ In this respect, numerous studies have reported that patients prefer a 6‐month subcutaneous injection to a weekly or monthly oral tablet.^58^, ^59^, ^62^, ^74^ Similarly, a monthly oral tablet is preferred over a weekly oral tablet. ^50^, ^52^, ^56^, ^57^, ^60^, ^63^, ^64^, ^65^, ^67^, ^68^, ^69^, ^73^

Efficacy and safety are also important attributes for patients.^51^, ^53^, ^54^, ^61^, ^66^ Nonetheless, some studies show that patients accept less effective and prolonged treatment if it does not cause adverse events.^59^ In addition to these issues, to reach an informed decision, patients demand further information about osteoporosis disease, drug‐specific detail (e.g., whether it is solid or liquid, or whether it should be stored refrigerated), and information related to healthy lifestyles (exercise and/or nutrition).^67^

## 5 CONCLUSIONS

This study provides an update on the current status of shared decision making in Osteoporosis treatment and the PDA currently available. The results highlight that patient preferences should be considered by physicians since they can impact adherence to the treatment and its efficacy. Currently, available PDAs can help to engage patients through shared decision‐making. Since the purpose of a PDA is to help patients in the decision‐making process there is certain information that must necessarily be included in the PDA. The information gathered in this review regarding the decision drivers may help to define which content should be included in a PDA.

## AUTHOR CONTRIBUTIONS

*Conceptualization*: Xavier Nogués, María Cristina Carbonell, Laura Canals, Luis Lizán, and Santiago Palacios. *Writing*: Luis Lizán. *Review and editing*: Xavier Nogués, María Cristina Carbonell, Laura Canals, and Santiago Palacios. Luis Lizán had full access to all of the data in this study and takes complete responsibility for the integrity of the data and the accuracy of the data analysis. All authors have read and approved the final version of the manuscript.

## TRANSPARENCY STATEMENT

Luis Lizán affirms that this manuscript is an honest, accurate, and transparent account of the study being reported; that no important aspects of the study have been omitted; and that any discrepancies from the study as planned (and, if relevant, registered) have been explained.

## Data Availability

Data sharing is not applicable to this article as no datasets were generated or analyzed during the current study.
